# Overview of Activities in the Field of Occupational Health and Safety during the COVID-19 Period Taken by Polish SMEs

**DOI:** 10.3390/ijerph20095630

**Published:** 2023-04-25

**Authors:** Marcin Olkiewicz

**Affiliations:** Faculty of Economic Sciences, Koszalin University of Technology, 75-343 Koszalin, Poland; marcin.olkiewicz@tu.koszalin.pl

**Keywords:** occupational health and safety, COVID-19, management, SMEs

## Abstract

The safety of work, employees, and clients of micro, small, and medium-sized enterprises (SMEs) is important because it is significantly related to the proper functioning and development of the entity and determines the decision-making process. The purpose of this publication is to show what actions aimed at increasing the sense of occupational safety and health during the COVID-19 pandemic were undertaken by Polish SMEs from the central Pomeranian region. The analysis of the literature most often presents the effects of the COVID-19 pandemic and the actions of governments in the field of protecting the public but does not present analyses of activities strictly taken by entrepreneurs. A survey was addressed to 300 business entities, of which 195 took part, determining the effectiveness rate at the level of 65%. Unfortunately, research shows that as many as 56% of the surveyed entities were negatively affected by the COVID-19 pandemic. Organizations used a number of safeguards aimed at increasing the sense of occupational health and safety, e.g., by using gels or liquids for disinfecting hands and surfaces during working hours (77%); regular cleaning and disinfection of equipment and workstations (84%); and maintaining social distance (76%). The analysis of the collected data covering the year 2021 indicates that this study should be treated as a survey study. This provides an opportunity to expand the area and scope of research. The presented research results indicate that, depending on the type of activity, as well as legal epidemic restrictions, SMEs increased the safety of employees and customers in different ways and with different tools during the development of the COVID-19 pandemic.

## 1. Introduction

The impact of the COVID-19 pandemic can be seen in every sector of the Polish economy as well as in the economies of other countries around the world. The pandemic forced numerous enterprises to completely or partially change their approaches to management and operation [1,2]. For some entrepreneurs, this has resulted in the expansion or improvement of financial conditions. However, for others, this led to the suspension or liquidation of activity. Epidemiological restrictions, in a significant way, have affected the conduct of business [3,4], as they have forced rapid and often radical changes in the processes of production (provision of services), organization of work, and, in particular, activities that guarantee the safety of employees and customers.

The issue of safety is very important, despite the difficulty of clear identification, because it is an interdisciplinary concept and is considered from various points of view, e.g., economic, legal, and IT [5,6]. It is essential that security determines the shape and functioning of any entity. Therefore, knowing that there are many discussions about the effects of the COVID-19 pandemic in various areas of the economy as well as in the social sphere or intervention policies of individual countries, reference is made to the issue of security taken up by SMEs because this is missing in the literature. It should be remembered that building occupational safety is a very important element of the proper functioning and development of a business entity. Furthermore, it should be assumed that ensuring occupational health and safety will directly or indirectly determine the quality of life, which is an important factor (trend) in the future development of globalization and the international economy. This is why it is so important to analyze the behavior of entrepreneurs: the main consequences of the COVID-19 epidemic are health effects that directly affect social and economic aspects.

The structure of this article is as follows: it begins with the presentation of the literature analysis, and then the approach to research is presented. In the next section, as part of the presentation of the research, individual actions taken by SMEs in the field of ensuring security are identified and analyzed. In the discussion, an attempt is made to present an overview of the actions taken to ensure occupational health and safety in the area of the organizational capabilities of SMEs. In this article, as part of the research, a comparative analysis was performed between individual enterprises (in terms of their size) and generalizations (for the entire surveyed population). As a result of the presentation of the conducted research, it was shown how entrepreneurs conducted a responsible security policy, guaranteeing the survival or development of their business in the era of the prevailing COVID-19 pandemic. At the end, the conclusions and limitations of this research are presented.

## 2. Literature Review

Safety is a vaguely defined term. Colloquially, it can be defined as a state of guaranteeing, e.g., peace of mind, independence, separateness, etc. It can also be defined as a continuous social process whereby individual entities seek to improve mechanisms to refine or create a sense of security [7,8]. Increasingly, the term security is becoming an important concept due to the prevailing war in Ukraine and continually developing COVID-19 pandemic. The impact of these determinants significantly affects the spheres of socio-economic life [9,10] and environment [11,12] of the countries of Europe as well as the world. Likewise, a significant influence can be seen in the field of business activities [13,14,15] as well as the formation of law. The concept of legal security refers to the constitutional principle of legal certainty, according to which an individual entity has the possibility to decide on their conduct based on a full knowledge of their rights and obligations [16]. However, it should be mentioned that the emergence of various threats may result in changes which, to a certain extent, may lead to destabilization or limitations in the short term. For example, one of the consequences of the emergence of the COVID-19 pandemic was the introduction of a series of restrictions of a socio-economic nature [17]. Starting with restrictions on free movement and assembly, up to restricting or completely banning certain forms of economic activity [5,18,19,20]. During the emergence and development of the COVID-19 pandemic, 57,658 legal acts came into force (including 21,677 (2019); 14,921 (2020); 20,960 (2021)), of which only 2011 acts concerned aspects of COVID-19 [21]. Mainly, the actions “imposed” by the Polish legislation directly related to the fight against the coronavirus pandemic but also introduced restrictions for enterprises and citizens as well as assistance programs that mitigate the effects of these restrictions. All the acts were intended to ensure occupational safety or build a sense of occupational health and safety among employers.

In accordance with the applicable legal acts, security and workplace hygiene is one of the priority issues in every developed country [22]. This concept applies to both occupational accidents and diseases as well as humanitarian considerations. It is beyond doubt that employed persons should be protected in the working environment. Article 66 of the Constitution of the Republic of Poland states that everyone has the right to safe and hygienic working conditions, and this right is part of social rights, which are implemented in practical terms [23,24]. In Poland, the legislator defines occupational safety and health (OSH) as a set of different types of factors that affect the health of an employee. Moreover, this definition refers to the elimination from the work environment of organizational, technical, hygienic, psychological, and economic factors that negatively affect the employee [25]. With regard to the labor law, the concept of occupational safety and health is defined as a set of legal guarantees aimed at securing human life and health in the work process [26]. This means that employers must create such conditions that not only protect the employee’s life at work but also protect them from the deterioration of both their physical and mental health. In conclusion, Polish OSH regulations are contained in legal acts that define the employer’s obligations towards the employee and are absolutely binding, as institutions have been established to effectively enforce the law in this respect [27].

The responsibility for the state of occupational safety and health in the workplace belongs to the employer. This responsibility does not include the duties of employees in the field of OSH and tasks entrusted to occupational safety and health services from outside the workplace. The employer is obliged to protect the health and life of employees by ensuring safe and hygienic working conditions, with the appropriate use of scientific and technical achievements. The employer is obligated to organize the workplace to provide safe and hygienic working conditions, to ensure compliance with OSH regulations and rules in the workplace, to issue orders to remove deficiencies in this respect, and to control the execution of these orders. Their duties also include responding to the development of a coherent policy to prevent accidents at work and occupational diseases. In addition, the employer shall ensure the implementation of orders, speeches, decisions, and decrees issued by supervisory authorities [27].

It should be noted that it is not only the employer who has obligations under the act regarding compliance with occupational safety and health. The employee is also obliged to comply with the law in this respect. In particular, the employee must be familiar with the OHS regulations and rules, participate in training and instruction in this aspect, and undergo a health check. It is particularly important that the employee should carry out their work in accordance with the current OSH regulations and rules as well as comply with the instructions and directions given in this respect by their superiors. They are expected to ensure the proper condition of machines, devices, tools, and equipment as well as order at the workplace and to use appropriate collective protection measures, assigned individual protection measures and work clothes as well as footwear in accordance with their purpose. In addition, they need to cooperate with the employer and superiors in the fulfilment of their duties concerning OSH. This is indispensable in a COVID-19 pandemic situation.

It is worth noting that changes in the Polish legislation during the COVID-19 pandemic introduced many positive actions (support) for entrepreneurs in Anti-Crisis Shield 4.0. The most important activities include a subsidy for the salaries of employees, a downtime pay for employees and entrepreneurs, the possibility of reverse accounting for the Personal Income Tax (PIT) and Corporate Income Tax (CIT) losses, microloans to 5000 polish zlotys, exemption from the Tax Office (ZUS), no need to obtain a new permit or declaration should changes be made to a foreigner’s working conditions, limiting the obligations related to the company’s social benefit fund, a subsidy for the salaries of employees who were not subject to downtime, or, as a consequence cancellation of a microloan up to 5000 polish zlotys.

**Figure 1:**
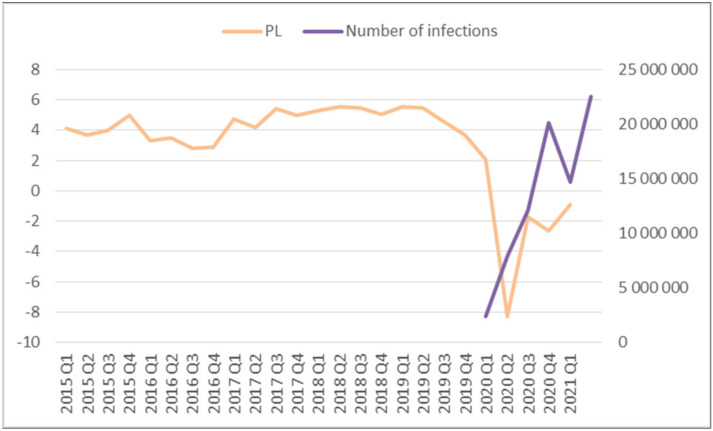
The evolution of GDP in Poland.

It should be remembered that the appearance of coronavirus at the turn of 2019–2022 triggered major changes in all areas of life for residents around the world. It has awakened new or increased needs in consumers and made business activity more difficult, restricted, and sometimes even terminated [28,29]. Numerous economic sectors, e.g., catering and tourism, could not provide their services comprehensively, resulting in significant macroeconomic changes, as presented in Figure 1.

The data presented in Figure 1 indicate that during the period of increasing COVID-19 infections, the country’s economic situation deteriorated so much that the Gross Domestic Product (GDP) index, as a measure of the size of the economy in the second quarter of 2020, fell by 2.8% compared to the previous year, where 4.5% GDP growth was recorded in 2019. According to the Central Statistical Office (CSO), GDP fell by 3.7% in 2020, with the degree of private consumption declining by 3%. The investment market also suffered, falling by more than 8%. The investment rate of the national economy in 2020 was lower than in 2019 (17.1%—2020, 18.5%—2019). Undoubtedly, the economic crisis caused by the COVID-19 pandemic and the resulting epidemiological situation, as well as the sanitary restrictions and the new problematic situation in which entrepreneurs found themselves, led to the occurrence of a recession in Poland for the first time in 30 years. The negative consequences of the prolonged epidemic among Polish entrepreneurs were visible in all areas of a business. Among other things, there were problems with financial liquidity, job reductions, and reduced sales and costs, as well as investment restrictions. A significant unplanned cost became the preventive measures taken by entrepreneurs to prevent the appearance and development of the pandemic among company employees. Taking care, in this difficult and specific period, of employees based on the real needs of direct protection measures, social as well as mental, could condition the preservation of adequate staff and limit the reduction of positions in the company. It was important for entrepreneurs, leaders, and managers at the head of employee groups and organizations to be very proactive and able to make rational decisions in times of dynamic changes in the market, including the scale of daily infection increases [30]. Responsible management, in terms of health and safety guarantees, enforced appropriate communication and implementation of new organizational solutions (e.g., remote working, rotational work, etc.), which were extremely important during the COVID-19 pandemic. It can be said that organizational management in the era of the COVID-19 pandemic took on a new dimension and had to respond to the needs of the epidemiological threat.

## 3. Materials and Methods

The data analyzed in this article are part of a research project aimed at identifying and evaluating activities in the field of dealing with COVID-19 by SMEs. The study was conducted in 2021 using a mixed-mode survey procedure, and CAPI and CAWI methods were applied. The research form consisted of 4 parts focusing on various aspects related to the functioning of enterprises during the pandemic. As part of the research, in the part covering the subject of this article, the focus was on the analysis of ensuring the safety of employees and clients in the process of work performed by organizations. The propensity of the organization to change the work process, as well as to provide employees with appropriate working conditions resulting from pandemic restrictions, was examined.

The aim of this research was to identify and evaluate the actions taken by entrepreneurs (SMEs) from the Central Pomeranian region in order to increase the sense of occupational safety and health during the COVID-19 pandemic. For the purposes of this research, a thesis was put forward: SME enterprises introduced sufficient changes in the work process, increasing or guaranteeing the sense of security and protection of employees and customers during the COVID-19 pandemic. The subjects of this research were enterprises (SMEs) from the Central Pomeranian region.

The unified and binding definition of a micro, small, and medium-sized enterprise in Poland is consistent with Recommendation 96/280/CE of the European Commission (1996) and includes employment parameters, the level of income, and the size of assets, taking into account dependencies with other entities.

**Figure 2:**
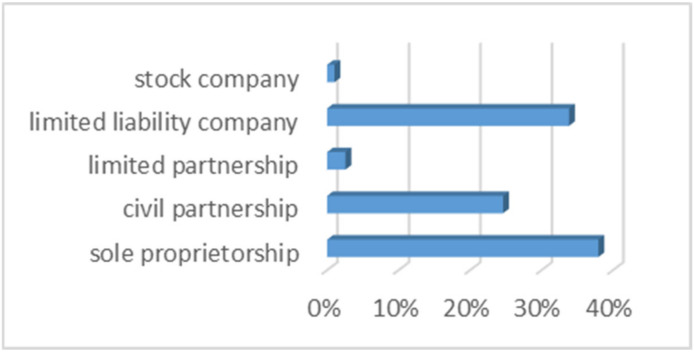
Forms of conducting business activity.

The main condition for the acceptance of the subject for this study was having employees and a location. The survey was sent to 300 entities. In total, 195 entities were surveyed, obtaining an efficiency ratio of 65%. The structure of participation in the survey of enterprises was 42% micro-enterprises, 33% small enterprises, and 26% medium-sized enterprises operating in various forms (Figure 2).

Business entities representing service (69%), production (17%), and trade (14%) activities took part in this study.

## 4. Results

Ensuring occupational health and safety during the COVID-19 pandemic caused the Polish government, in response to the WHO-announced pandemic, to introduce several restrictions and limitations to limit the spread of the SARS-CoV-2 coronavirus. The government, in order to protect Polish entrepreneurs, implemented a law on specific solutions related to counteracting, preventing, and combating COVID-19 and the crisis situations caused by it, the so-called ‘anti-crisis shield’. This law and subsequent anti-crisis shield were based on five pillars: protecting jobs and workers’ security; financing entrepreneurs; health care; strengthening the financial system; and public investment. They were designed to defend the country from the crisis and, in particular, to help organizations to survive. The use of government assistance has even allowed some organizations to grow, as Figure 2 confirms.

**Figure 3:**
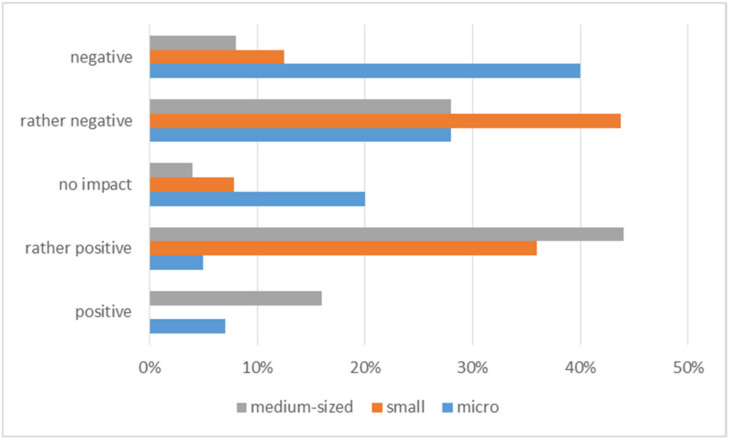
Assessment of the pandemic’s impact on the functioning and development of the organization.

**Figure 4:**
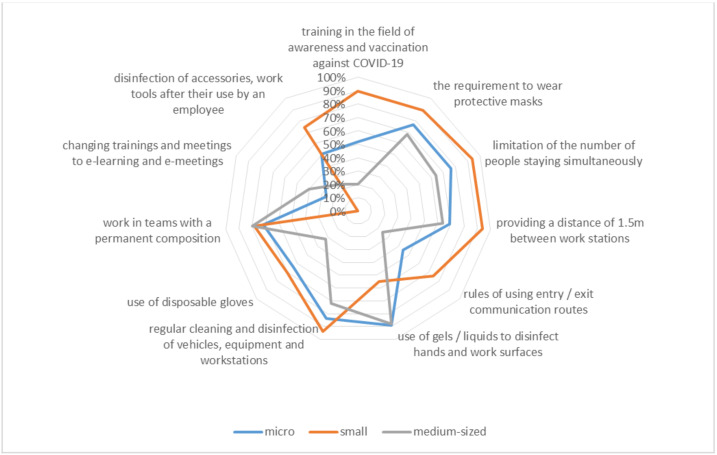
Actions taken by the organizations.

The analysis of the data presented in Figure 3 shows that the distribution of opinions is ambiguous. The graph shows that medium-sized enterprises coped better in the difficult pandemic situation than micro-enterprises. As many as 60% of the surveyed medium-sized enterprises positively assessed the impact of the COVID-19 pandemic on the organization’s ability to function and develop. Of course, all activities had to be carried out with rigor to ensure work safety. Figure 4 shows what actions organizations have taken to protect employees from the risk of contracting COVID-19.

Figure 4 shows that the largest number of preventive measures to increase work and employee safety were implemented by small enterprises and the least by medium-sized enterprises. Such actions could have been caused by a change in the way work was performed, which did not require the implementation of additional preventive measures. It is worth noting that entrepreneurs undertook various actions (Table 1) to survive this difficult pandemic time.

**Figure 5:**
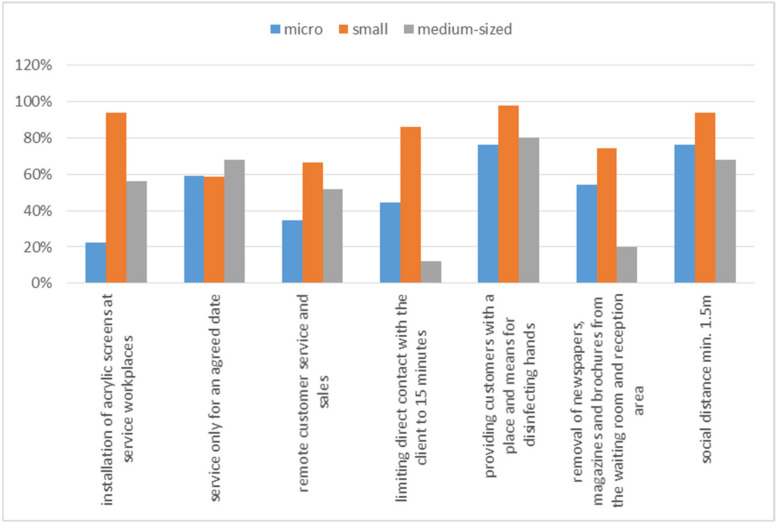
Activities in the area of ensuring customer safety.

From the employee’s point of view, only “guaranteeing employee safety” is insufficient. The conducted research also included the identification of the safety of potential customers, who also needed to be protected against a potential threat. Figure 5 presents the activities undertaken by entrepreneurs in the area of ensuring customer safety.

Figure 5 shows that also in this case, small enterprises tried to secure the safety of customers the most. Of course, some of the preventive actions implemented in relation to employees could also directly apply to customers. However, it was the skillful and responsible actions of entrepreneurs that were supposed to lead to increased security and optimization of activities so that the functioning of the organization was not endangered and so that the organization could take advantage of emerging opportunities in the era of the COVID-19 pandemic.

## 5. Discussion

The analysis of the presented data shows that Polish organizations complied with government guidelines by implementing various activities in organizations aimed at increasing occupational health and safety [22]. Despite the growing epidemiological threat (Figure 1), entrepreneurs at SMEs tried to use protective measures to maintain or develop the organization. Undoubtedly, pandemic restrictions and the effects of the development of COVID-19 in the world and in Poland had a negative impact on business. However, it can be noticed that 8% of respondents positively assessed the existing situation. This may mean that these organizations were able to adapt faster to the emerging epidemiological changes and take advantage of the opportunities and assistance provided by the Polish government. Regardless of the method of managing the organizations adopted, employers took various actions during the development of the pandemic, also resulting from legal acts, aimed at ensuring the safety of employees.

The research results indicate that among the most frequently used forms of counteracting the effects of the COVID-19 pandemic, employers at SMEs indicated the use of gels or liquids for disinfecting hands and work surfaces by employees (77%) and regular cleaning and disinfection of vehicles, equipment and workstations (84%). Interesting and important phenomena used by employers to increase the safety guarantees for employees were “forcing” employees to wear protective masks (79%) and limiting the number of people staying in one place at the same time (79%). The vast majority, as much as 55% of the surveyed entrepreneurs at SMEs, did not decide to take action related to changing training and meetings in a direct (traditional) form to e-learning or e-meetings. This situation may result from the number of students participating in such training and the skillful organization of the training place while maintaining a safe distance.

Entrepreneurs also paid a lot of attention to potential customers. Ensuring their safety could guarantee, in some cases, functioning and generating income [30]. It was especially visible in shops, restaurants, pharmacies, or medical practices. The Polish state also took care of the safety of customers during service. The regulation issued by the Polish government included a number of forced restrictions to which entrepreneurs had to adapt. The most frequently used changes by entrepreneurs are shown in Figure 5. The two most frequently chosen safety measures by the surveyed entrepreneurs at SMEs in relation to the customer were maintaining social distance (80%) and providing disinfectants (84%). On the other hand, the two most frequently used security measures for customers by the respondents were the installation of acrylic screens (54%) and remote service (61%). In the case of acrylic screen installation, in as many as 26% of cases, entrepreneurs indicated that such an obligation did not apply to them. This means that 18% of the respondents, although they could use this form of security, consciously did not do so.

**Figure 6:**
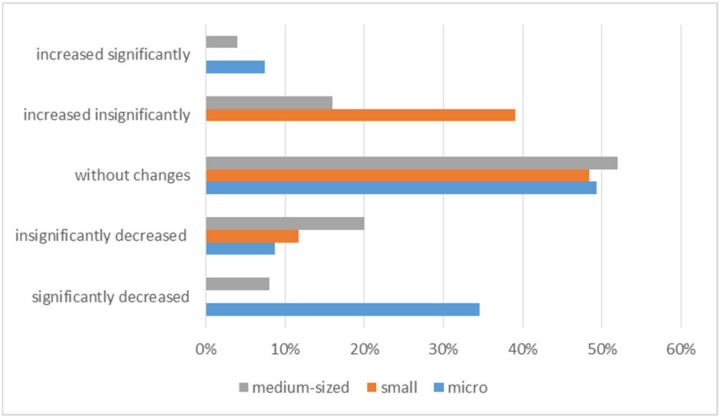
Employment level.

The analysis of the effects of the COVID-19 pandemic, presented in many publications [3,4,5,13,18,28,29,30], in terms of the structure and size of employment in macroeconomic or regional terms, indicates significant changes. This may result from the need to take measures to minimize the costs of maintaining the organization in the market, aimed, for example, at implementing changes in the way of working. Changes in the manner in which work was performed were also implemented in the surveyed organizations. Despite this, only 50% of the surveyed SMEs managed to maintain employment; in 29% of the entities this state decreased, and in 21% of the respondents it increased. Such results may indicate that the COVID-19 pandemic played an important role in shaping personnel policy. However, it should be noted that despite the negative impact of COVID-19 on the functioning of business entities, some of them achieved benefits in the employment structure (Figure 6).

**Figure 7:**
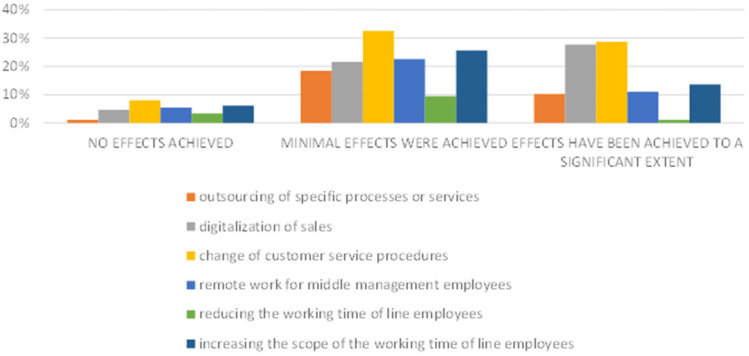
Implemented changes and achieved effects in SMEs.

Such results may indicate that the COVID-19 pandemic was not such an important determinant of maintaining the employment structure. However, a detailed analysis of the data in Figure 6 shows a slightly different result, which means that such results cannot be generalized. This is particularly visible in the assessment of employment in micro and medium-sized enterprises. This means that, despite the protective measures taken by the government and the measures taken by entrepreneurs within the available funds, conducting business and planning development was difficult. It is worth noting that entrepreneurs, in order to retain employees and protect them from the effects of the pandemic (e.g., health), in addition to preventive measures, implemented other measures, e.g., in changing the organization of work or the functioning of entities (Table 1). The research shows that these actions had different effects across SMEs (Figure 7).

## 6. Limitations

A key limitation of this study is the method of data collection. This is because some companies were unwilling to respond. It is worth noting that the difficulties in running a business resulting from the emergence and development of the COVID-19 pandemic were also identified, assessed, and experienced differently by entrepreneurs, which resulted in (despite all) a low effectiveness of the survey and a significant dispersion of data.

The restriction was also related to

- limiting the study to one region (Central Pomerania);
- the short period of the pandemic, 2020–2021 (which was used for the analysis).

These two limitations may indicate pilot studies. Therefore, in the future, it will be worth analyzing the activities undertaken by entrepreneurs in the long term and in entities from various regions of Poland or from the entire country. It will also be worth conducting a comparative assessment of activities carried out by entrepreneurs from different EU countries. In particular, by analyzing what actions have been maintained during the “pandemic” period and whether government actions, in a systemic way, maintain (support) the need to maintain epidemiological restrictions. It is also worth analyzing in the future how the work safety developed in the COVID-19 era affected statutory changes regarding work (e.g., working conditions, forms of work, methods of employment, etc.), including in individual branches of the economy.

In summary, the presented research results may constitute the basis for further, broader, and in-depth research in the area of the impact of the COVID-19 pandemic on the economy.

## 7. Conclusions

Changes resulting from the emergence and development of the COVID-19 pandemic and associated restrictions have significantly affected the way organizations operate and perceive workplace safety. All surveyed organizations consciously followed the recommendations of the Polish government and the WHO to implement measures to reduce or eliminate emerging new pandemic outbreaks. For most of the surveyed organizations, this meant implementing safety procedures and adapting to a rapidly changing environment. The presented research results confirm the thesis that Polish SME enterprises introduced sufficient changes in the work process, increasing or guaranteeing the sense of security and protection of employees and customers during the COVID-19 pandemic. Despite responsible management during the pandemic, respondents stated that:

1. The pandemic had a negative impact on business.
2. The implemented changes in the organization of work were most effective in the area of digitalization of sales and changes in customer service procedures.
3. For 50% of the surveyed entities, the employment status has changed. For 29% of respondents, employment status has decreased.
4. The measures implemented to guarantee occupational health and safety resulted from the financial and organizational capabilities of the entities.

Difficulties and government restrictions forced, where necessary and sometimes possible, the introduction of increased security measures, which exposed organizations to increased costs of conducting business. Research has shown that organizations have counteracted global recessionary trends in different ways, with varying degrees of success. Nevertheless, they tried to provide appropriate conditions, guaranteeing a sense of job security for employees and customers.

As part of the conducted research, the following recommendations can be indicated:

1. The need to monitor and improve occupational health and safety supervision procedures.
2. Building awareness among employees of the existing epidemiological threats.
3. Continuous verification of the use of measures to increase protection and safety at work.

## Figures and Tables

**Table 1:** Implemented changes and achieved effects.

Changes/Result	Not Implemented			No Effects Achieved			Minimal Effects Were Achieved			Effects Have Been Achieved to a Significant Extent		
	Micro	Small	Medium-Sized	Micro	Small	Medium-Sized	Micro	Small	Medium-Sized	Micro	Small	Medium-Sized
**outsourcing of specific processes or services**	90%	63%	48%	0%	3%	0%	7%	16%	40%	2%	19%	12%
**digitalization of sales**	77%	19%	32%	4%	0%	12%	7%	34%	28%	12%	47%	28%
**change of customer service procedures**	65%	8%	4%	2%	12%	12%	12%	43%	52%	20%	38%	32%
**remote work for middle management employees**	88%	38%	48%	2%	4%	12%	2%	47%	24%	7%	12%	16%
**reducing the working time of line employees**	83%	92%	48%	5%	4%	0%	10%	4%	16%	2%	0%	0%
**increasing the scope of the working time of line employees**	85%	59%	0%	7%	3%	8%	5%	34%	48%	2%	4%	44%

## Data Availability

Data are contained within this article.
